# When the law makes doors slightly open: ethical dilemmas among abortion service providers in Addis Ababa, Ethiopia

**DOI:** 10.1186/s12910-019-0396-4

**Published:** 2019-09-05

**Authors:** Emily McLean, Dawit Nima Desalegn, Astrid Blystad, Ingrid Miljeteig

**Affiliations:** 1 Department of Global Public Health and Primary Care, University of Bergen, Kalfarveien 31, 5018 Bergen, Norway; 2 College of Health Science, Addis Ababa University, Addis Ababa, Ethiopia

**Keywords:** Abortion, Abortion service providers, Moral distress, Abortion law, Ethical dilemmas, Empirical ethics, Unsafe abortions, Women seeking safe abortion, Inequalities

## Abstract

**Background:**

In 2005, Ethiopia changed its abortion law to curb its high maternal mortality. This has led to a considerable reduction in deaths from unsafe abortions. Abortion is now legal if the woman’s pregnancy is a result of rape or incest, if her health is endangered, if the fetus has a serious deformity, if she suffers from a physical or mental deficiency, or if she is under 18 years of age. The word of the woman, if in compliance with the law, is sufficient to qualify for an abortion. In this context, where the law makes the door slightly open, health workers become important in deciding who gets access to safe services and who doesn’t, thus creating considerable ethical dilemmas.

**Methods:**

The objective of this study was to explore abortion service providers’ personal experiences and reflections, perceptions of the abortion law, and ethical and dilemmas that arise. Data collection took place from March to May 2016 in Addis Ababa, at different health clinics providing abortion services. Thirty in-depth interviews and three focus group discussions were conducted with 41 abortion service providers at governmental and non-governmental clinics. Content analysis was drawn upon in the interpretation of the findings.

**Results:**

When working in a context where the law has slightly opened the door for abortion seeking women, the health workers describe conflicting concerns, burdensome responsibilities, and ambiguity concerning how to interpret and implement the law. They describe efforts to balance their religious faith and values against their professional obligations and concern for women’s health and well-being. This negotiation is particularly evident in the care of women who fall outside the law’s indications. They usually handle ethical dilemmas and decision-making alone without guidance. Moreover, many health workers face a stigma from fellow colleagues not performing abortions and therefore keep their job a secret from family and friends.

**Conclusions:**

Health workers in Ethiopia experience ethical dilemmas trying to maneuver between the abortion law, their personal values, and their genuine concern for the health of women. More research is needed to further explore this.

**Electronic supplementary material:**

The online version of this article (10.1186/s12910-019-0396-4) contains supplementary material, which is available to authorized users.

## Background

As Richard Horton, the editor-in-chief of *The Lancet*, stated in 2016, *“*There aren’t too many taboos left in global health [...] but abortion remains a forbidden word,” pointing to the high number of women still dying from unsafe abortions globally [1]. According to the World Health Organization (WHO), each year between 4.7–13.2% of maternal deaths can be attributed to unsafe abortion [2]. Abortion-related deaths have been described as a “silent pandemic” and a neglected sexual and reproductive health issue [3].

Globally, there is a broad continuum of legal categories for abortion. Some countries allow abortion on a woman’s request with no requirement for justification, some claim specific grounds, while in other countries there is an uncertain prohibition where laws prohibit unlawful abortion but do not specify any lawful grounds, in a few countries abortion is prohibited on all grounds [4]. The laws have been debated and changed throughout history with different motives behind what they are meant to regulate [5].

Even where abortion laws are relatively liberal, like in the USA and South Africa, many women may still struggle to access the service [6, 31]. Distance to health-care institutions, the strict regulations of these institutions, privacy at the clinics, the cost of the procedure, and the availability of health workers willing to provide abortion services may all create access barriers [6]. The latter point is important as the WHO has pointed out how the lack of health workers providing abortion services is a major barrier for women’s access to safe abortion [7]. Finding health workers willing to provide abortion care can be difficult, especially in settings with restrictive abortion laws, and where the profession is associated with shame, and the abortion service providers are facing stigma and discrimination [8].

Therefore, learning more about how abortion service providers perceive and adhere to the laws and abortion policies, how they view their own roles, and how they make abortion-related decisions is vital to gaining a better understanding of the complexities of access barriers to abortion services. In particular, enhanced knowledge of health workers’ perceptions and experiences may guide the field in how to develop and facilitate the training of and support for professional abortion service providers.

In a review of health workers’ attitudes and perceptions towards induced abortion in Sub-Saharan Africa and Southeast Asia, Loi et al. found that local culture and social norms played a crucial role in shaping abortion service providers’ perceptions and conduct around abortions [9]. In addition, abortion service providers were generally found to be uncertain about the legal status of abortion in their countries [9]. A study of abortion service providers in Zimbabwe found that the community perception of abortion as morally wrong led to abortion service providers having a negative view of women seeking abortion services [10]. Being an abortion provider can be difficult, as demonstrated in the Aniteye et al. study from Ghana where the stigma associated with abortion led to abortion service providers being reluctant to offer the service for fear of community repercussions [11]. In Senegal, Suh found that some abortion service providers would manipulate medical records to conceal illegal abortions [12].

**Fig. 1:**
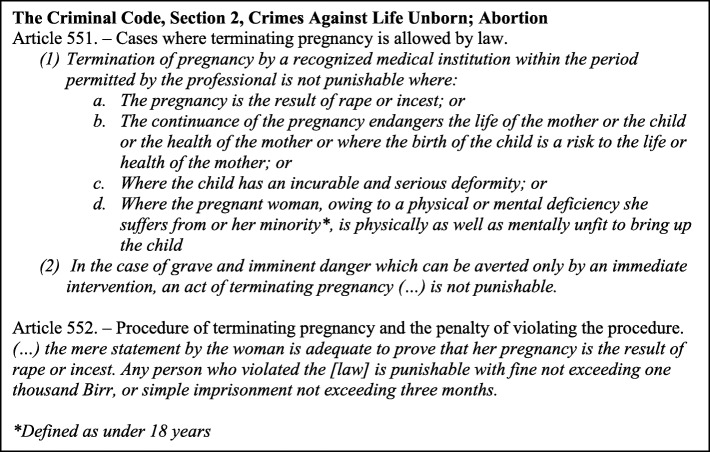
Extract from The Criminal Code of The Federal Democratic Republic of Ethiopia

Deaths from unsafe abortions were identified as the main reason behind Ethiopia’s extremely high maternal mortality ratio of 968 per 100,000 live births in the 1990s [13, 14]. In an effort to curb the high number of women dying from unsafe abortions, the Ethiopian abortion law was changed in 2005, expanding the number of grounds for which lawful abortion could be provided as detailed in Fig. 1 [15, 16]. A key innovation, which makes the Ethiopian law unique in an African context, is that health-care providers are obliged to accept without question a woman’s word regarding rape, incest and her age.

Information about abortion in Ethiopia is scarce. The latest data estimate that of the 620,300 abortions conducted in 2014, 47% were considered unsafe [17]. Many women and girls continue to face substantial difficulties accessing safe abortion services [18]. Muzyen et al. found that, despite the change in the law, many young women still do not have sufficient information about the law and their rights to a safe abortion [19].

Shortly after the abortion law was changed, the Ethiopian government issued clinical guidelines for safe abortions [15]. The guidelines define abortion as up to 28 weeks of gestation. Abortion can either be done medically using Misoprostol and Mifepristone or surgically where manual vacuum aspiration (MVA) is the preferred method. A second trimester abortion is defined as over 12 weeks of gestation and can only be conducted at hospitals by specialized doctors.

With the unique phrasing in the Ethiopian abortion law of a woman’s word being sufficient to gain abortion, many countries look to Ethiopia for inspiration to reform their abortion law [17]. Ethiopia thus emerges as a particularly interesting site in which to study health workers’ perceptions and experiences of abortion-related work. However, to date, the Ethiopian abortion service providers’ perspectives have not been studied in-depth. The objectives of the study were to explore Ethiopian abortion service providers’ reflections of their work, their perceptions and interpretations of the abortion law, and the potential ethically challenging aspects of their work.

## Methods

### Study setting

The study was conducted in Addis Ababa, the capital city of Ethiopia, a melting pot of different religions and ethnicities. The city stands out as the richest and most developed area in the country [20, 21]. Administratively, the city is divided into 10 sub-cities. The total fertility rate of 1.5 is half the national average [21]. Further, the city has the highest contraceptive coverage in the country at 50,1% usage of modern contraceptive methods [20]. Addis Ababa also has the highest registered abortion rate in the country estimated at 92 per 1000 women aged 14–49 years [17].

Investigation was carried out at four public health centres, two hospitals, and five non-governmental organizations (NGOs) clinics providing abortion as well as other health services such as family planning, post-abortion care and other gynaecological services. The abortion service providers met the women seeking induced abortion either through an elective appointment or at the emergency room.

The various health facilities provided abortion services in different ways. At the public health centers, abortion was provided free of charge and was open Monday to Friday. At the NGO clinics the price varied with some providing abortion to a reduced price to poor women. They were also open Monday – Friday. The hospitals also provided free abortion services, but there women could seek an abortion by going to the gynecological emergency room which was open 24 h all days. There were commonly one or two nurses working as abortion service providers, although at the hospitals and NGO clinics a doctor often worked alongside the nurses with providing abortion services or would be called for when encountering challenging cases, such as second trimester abortions which only the doctors could perform. Commonly, the head nurse or doctor assessed the woman’s eligibility through a consultation where they would either accept or reject her request for an abortion. Before the abortion was conducted the doctor or nurse and the woman wishing to obtain safe abortion signed a consent form.

The size of the study sites varied, at the public hospitals induced abortions were performed at the minor gynecological emergency room which consisted of three to four beds, while the health centers only had one small private room with one bed used for the procedure. The NGOs either had a whole department only for abortion services or it was mixed with general gynecological services. They always had several private rooms to perform the procedure. No official statistics on the abortion caseload per clinic was available but through the interviews we got to understand that the health centers performed the least abortions with three to ten per month, the hospitals seemed to perform more especially since second trimester abortions all had to be performed here. Though most abortions seemed to be performed at the NGO clinics who stated that they performed several hundred abortions per month. All clinics at times experienced a lack of staff and medicines*.* At all the clinics visited during the study, surgical abortion was reported to be more common than medical abortion, though this picture was reported to be changing.

### Data collection

Data collection took place from March to May 2016. Participants were included if they worked with any aspect of induced abortion services provision at the time of the study including either provision of pre-abortion information and counselling, provision of abortion pills or manual vacuum aspiration (MVA) and post-abortion care. They were recruited by the first author (EM), with assistance from the co-author (DD), who works as a gynecologist in Addis Ababa. At the initial stage of the recruitment purposive sampling was used to ensure the inclusion of participants from different abortion service providing institutions and from different cadres of health workers. Later snowballing was employed to identify new participants. A total of 31 in-depth interviews (IDs) were conducted, of which three were follow-up interviews made to clarify important emerging topics. One interview took place with two people as the attendance of them both was requested by the participants. In addition three focus group discussions were conducted (FGDs) with two groups of five and one group of three participants.

The participants were between 23 and 42 years old and had between a few months and 15 years of experience working with all aspects of abortion service provision from actually inducing the abortions to taking care of women undergoing an induced abortion. Of the abortion service providers, 19 were male and 23 were female. The majority, 24 participants, worked as nurses with nine having additional training in midwifery, five worked as doctors, three as health officers, three as medical students that had training in induced abortion and one pharmacist. The majority, 16 participants, considered themselves Orthodox Ethiopian Christian, three as Protestant, five as Christian, two as Muslims and six were religious without further specification. It must be noted that information about age, profession, years of working experience and religion was not obtained from all the participants as abortion is a sensitive topic and it was not always appropriate to ask about this. An additional file with more details about the characteristics of the study participants is provided (see Additional file 1).

A semi-structured interview guide was employed. To ensure that the questions were as relevant as possible to the context, adaptations to the guide were made during the course of the fieldwork to incorporate emerging issues. The majority of the interviews were conducted in English by the first author (EM). The focus group discussions and seven individual interviews were conducted in Amharic by a research assistant trained in qualitative methodology (with EM present). The interviews took place in separate rooms at the health institution during breaks or after working hour. Two interviews were conducted at a restaurant following the participants request. Interview participants were recruited until a sense of saturation was reached, that is, when no major new themes emerged [22]. All interviews and focus group discussions were audio recorded.

### Data analysis

Preliminary analysis started during the fieldwork through a process of continuous reflection on the information emerging during the interviews. Moreover, field notes with reflections were written on a weekly basis. The interviews in English were transcribed by the first author (EM), while the Amharic interviews were transcribed and translated by a professional Amharic-English translator. Based on the patterns identified while in the field, content analysis was drawn upon for systematic analysis post fieldwork [23]. EM read through the full data set several times, getting a sense of the overarching themes, and divided the material into several meaning units, which were then discussed with the co-authors. The content within each unit was then condensed, coded, and sorted into categories. Finally, the categories were turned into generalized descriptions of the most common dilemmas, judgments, and reflections emerging from the material. These were supported by specific verbatim statements from the interviews. NVivo11 software was used in the process of coding and organizing the data [24].

### Ethical approval

Ethical approval was provided by the Initial Review Board and by the Institutional Review Board at Addis Ababa University, College of Health Sciences. Ethical approval was applied for from Regional Ethical Committee of Norway (REK), but the study was considered to be outside their mandate. General research ethics principles of anonymity, confidentiality, and rights of withdrawal without consequences were followed. The interviews took place in rooms where privacy was ensured and all the recordings were kept safe on a closed file in the authors computer. In cases where the participants had to travel to be part of the study, compensation for the travel was provided. All participants signed a consent form.

## Results

The study revealed that health workers providing abortion services experienced diverse and numerous challenges in their work. Ethical dilemmas were reported concerning abortions and related decision-making. How the abortion service providers perceived and interpreted the abortion law seemed to influence the way they handled and coped with these dilemmas.

### “The law might be clear, but the reality is not”

The current abortion law and clinical guidelines were well-known among the health workers. They all stated that they followed the recommendations and often referred to them during our interviews, but they also explained that they experienced a gap between the law and the working reality at the clinic, which continuously forced them into ethically challenging situations. Three main dilemmas were described by the participants; (1) should abortion be provided or not (2) should they accept lies or not, and (3) should they accept all kinds of reasons or not. Each of these scenarios is discussed below.

#### Should abortion be provided?

The health workers in the study described various dilemmas arising when a woman is not eligible for an abortion according to the grounds stipulated in the law. They worried that if they refused the woman a safe abortion, she would end up trying to self-induce the abortion or seek abortion from an unskilled provider. Health workers were aware, and some had personal experiences, of how refusing care to a desperate woman seeking abortion could have harmful and even deadly implications. A health worker explained this dilemma as follows:

*“Her case was not listed within one of the rules (that allows one) to get an abortion. This became an obstacle for me of whether to conduct an abortion or not... If I refused to assist her, I knew she would take desperate measures.” (17, ID)*

One health worker had experienced a case where refusal led to the patient dying from unsafe abortion and recalled how such the experience had transformative impact on his attitude towards abortion.

*“I refused to provide her with the services since her reason was legally not sufficient to get an abortion. Three days later I was on a night shift and she came to the health station with an ambulance after having tried to abort on her own. She was bleeding badly when she arrived and soon her life passed away. We lost this woman’s life on our watch and this should teach us a great lesson.” (28, ID)*

The feeling of being responsible for her death made him see abortions differently. Similar experiences were reported by several health workers who reflected on the situation before the 2005 law was implemented. They presented detailed memories of how emergency rooms were filled with women suffering serious and life-threatening complications from unsafe abortions.

#### Handling lies

Another challenge arose when health-care workers believed that the woman was lying about her reason for having an abortion. According to the abortion law and guidelines, health-care providers are to accept a woman’s word of rape, incest and age. Yet many found this unpleasant and upsetting, which in turn made them confused about the ethically correct way to handle the challenge. They explained that they felt the women were trying to trick them into getting an abortion.

*“They will claim to be raped, but you can just see … From their physical stature, the emotional and the psychological appearance, you will know that it’s not the case, but you still would have to provide the service. That’s one of the dilemmas that you face.” (6, ID)*

Participants revealed that they handled these dilemmas in different ways, depending on their views about the women’s legal arguments, as well as on considerations of the potential consequences a rejection could have. Several health workers described situations where they slightly modified the woman’s request so that she became eligible for the abortion services. They were open to ‘stretching’ the interpretation of the law and used an approach one informant described as *“*doors that are slightly open*.”*

*“Sometimes, the legal part has a slightly open door. We use such doors to help the clients, but if medical problems happen I will be courted [sent to jail]. So even if the door is slightly open it is challenging for us. We will try to help them, but lifesaving is not easy. Sometimes we do take a risk to help them.” (22, ID)*

#### Assessing the legitimacy for abortion

The expressed risk-taking of the abortion service providers, their particular interpretation of the law, and whether they made a modification to a woman’s request, depended on how they perceived the woman’s immediate need, the long-term consequences of the decision, and whether or not she was perceived to have a “good enough” reason for an abortion. Many of the abortion service providers used the term “reasonable abortions.” What in their view would not qualify as an acceptable reason for an abortion was referred to as an “unreasonable abortion.” Such an assessment did not always follow the lines of the law.

Many of the abortion service providers felt particularly sympathetic towards young women, especially students and poor young women. They expressed that they felt responsible for helping such women as an abortion could prevent them from dropping out of school, being ostracized by their communities, or falling into even deeper poverty. Confronted with such scenarios, the large majority of the abortion service providers in the study found it difficult to refuse assistance to these women despite their not being considered as legally eligible. Such categories of women would hence often be described as ‘reasonable’ women wishing to obtain safe abortion, as they were seen as in dire need of help.

*“Sometimes I’m satisfied with what I’m doing in the abortion case. For some clients, maybe they are very poor, the poorest. Most people with unwanted pregnancy are the poorest ones.”(23, ID)*

In contrast were the women whom the abortion service providers often did not perceive to have a good enough reason for an abortion. These were, in particular, married women as well as women thought to be wealthy. They were described as women trying to cheat the system by saying that they had been raped or who were untruthful about their age. Such women were commonly described as liars or cheaters. Some abortion service providers tried to convince them to go on with their pregnancy with counselling sessions, while others refused altogether to provide them with the service.

*“If they are reasonable, I have to help them. If they are not reasonable, I have to reassure them to continue the pregnancy.”(22, ID)*

*“If she is married that’s difficult, that’s not good. Then sometimes we ignore her.” (26, ID)*

Moreover, the health workers’ perceptions of the abortion law seemed to influence their assessment of whether an abortion was reasonable or unreasonable. Health workers who viewed the law as being too restrictive often found that women had good and reasonable reasons for an abortion. They appreciated that the law enabled them to help more women to get an abortion, and would use “the slightly open door” policy as a justification for providing the service. However, health workers who viewed the law as being too permissive or liberal were quicker to assess women’s explanations as unreasonable. They would often question women thoroughly in attempts to uncover whether or not they were lying about the reason for seeking an abortion. Some of these participants felt strongly that the existing law was too liberal and thought that abortions should only be accepted to save the life of a woman, as was the case prior to the change in the law. Religious arguments of abortion being a sin were strongly present within this line of reasoning.

*“If it is rape I think it’s better [for her] to keep the pregnancy, because I’m a Protestant, and I think it is a sin to terminate a pregnancy that is alive.”(5, ID)*

### “Am I conducting a crime?”

Even though most health workers accepted that some women were eligible for an abortion, many felt that the very act of carrying out the abortion was difficult. Standing in front of a woman and administering the drugs, or using the surgical devices to remove the fetus, provoked dilemmas related to their religious beliefs, perceptions about life, and local societal norms. They questioned whether abortion was ethically right or not. This dilemma was often expressed in terms of “feeling something awkward” and in feelings of “shame” and “wrongdoing.”

*“I also feel somewhat awkward while I am doing my job. However, the mothers who come here seeking abortion would probably go to a much worse place if I told them that I would not provide them with the abortion services due to my feelings or my religion.” (31, FGD)*

All the participants described themselves as religious, and this influenced their perception of abortion. The religious teaching that abortion is sinful made it difficult for many to fully accept the nature of their work. Some health workers explained that they would pray after having conducted an abortion and were worried that their God would not forgive them.

*“Our religion does not support conducting abortions and states that it is a sin. Though we do it to help mothers who are in need, it makes us feel bad towards ourselves.” (31, FGD)*

Some expressed that abortion was in conflict with the medical principle of “do no harm,” and felt they were ignoring this principle when performing abortions.

*“It conflicts with my morals, my beliefs. I was taught in school to do no harm.” (6, ID)*

Furthermore, the health workers explained that, according to the cultural norms in Ethiopian society, abortion is taboo and is viewed as a crime by many. As community dwellers and participants in the society, they thus had to strike a balance between the social norms surrounding them and their work. Conducting abortions was experienced as a moral judgment where the health workers had to weigh the woman’s need of help against what many considered as taking the life — or even killing — an unborn child.

*“Sometimes it is ethically too hard, because there is a challenge with the societal and religious cultures. Some may say that professionals are killing the baby. So you may feel this when you are interacting with religion and society.” (3, ID)*

### “I don’t tell them I conduct abortions”

While the health workers interviewed revealed a high commitment to their work and a conviction that they were saving women’s lives and preventing suffering, the religious anguish and the stigma and marginalization they experienced from colleagues led to frustration and burnout. Many felt that they were standing alone with difficult decision-making and missed a support network.

*“There is no support group to uplift our morals and skills, we are just expected to deliver a given number (of abortions), and I think this itself has a moral impact.” (32, FGD)*

Some described how they would jokingly be called “anti-generation” or “child killer” by colleagues who were not performing abortions. The negative perceptions of health workers providing abortion services caused many to hide the nature of their work from family and friends. Some said they could be kicked out of their home if their family heard that they performed abortions. Others said they were likely to lose dear friends. Many, therefore, came to disguise their work.

*“When people ask me what my job is I tell them that I am a delivery nurse. I don't tell them that I conduct abortions. My family members are church people so if I tell them that I conduct abortions they will be very angry. I even fear that they might expel me from the house if they find out that I conduct abortions.”(29, ID)*

### “A way to save our clients’ lives”

In such difficult work-related contexts, the large majority of the study participants found strength in religion and in the perception that they were doing good by helping the women. Several referred to the conviction that they were sure that God would understand or forgive them, given their underlying motive of saving lives.

*“Step by step, you’ll accept (the work), because on one hand there are religious and cultural influences, and on the other hand we see this vulnerable group of helpless people.”(14, ID)*

*“I see abortion as a way to save our clients’ lives because we are protecting these women. If we provide a safe and sound abortion, we are rectifying their lives. For these reasons, I don't see being engaged in abortion-related services as a sinful job.” (24, ID)*

It should be noted that, for a small minority of our study participants, working with abortion services did not emerge as particularly problematic. It was perceived as a job that would bring money to the table or as part of the right of a woman to decide over her own body.

*“So I don’t have a chance to reject the work because I want to live and earn money ( … )” (23, ID)*

*“The rule should support every woman’s right to get an abortion. It does not have to restrict some women's right and approve others’ rights” (24, ID)*

Having experienced the positive impact their work had on women’s lives, many of the abortion service providers indeed felt that their job was worth the stress and the risk. One health worker particularly mentioned an example of having helped a young student who had been raped. Because she was provided with an abortion, the student managed to finish her education. Such experiences helped the health workers to justify the day-to-day challenges of their work.

*“To me, they say ‘thank you so much, I have completed my university education.’ When you have experienced this, you become very happy to help them.”(25, ID)*

## Discussion

### Providing abortion within the scope of the law

Our findings indicate that the abortion service providers made a substantial effort to follow the abortion law and that they attempted to slightly stretch the interpretation of the law in certain instances and to make such bending of the law ethically justifiable to themselves.

The Ethiopian law is unique in the way it opens up for abortion if certain criteria are fulfilled, and that the women’s word is enough to ensure an abortion. Although the law remains within the country’s Penal Code it has nevertheless left “the door slightly open” as a participant phrased it. Our study shows that also the variations found among service provider discretion relating to a woman’s eligibility for abortion under a particular legal ground, influence if the door is indeed open or not. The outcome of the abortion service provider counselling, their eligibility investigation, and their assessment of a woman’s reasons as ‘reasonable’ or ‘unreasonable’ indicate how women’s access to safe abortions were dependent on the health workers’ particular judgments and values. Women who were perceived as rich or married were, more often than the young, poor, and students, seen to have unreasonable or illegitimate reasons for an abortion. The example of married women is particularly noteworthy as marital rape is not recognized in the Ethiopian law, thus creating a sense of ineligibility for abortion among these women [25]. This idea can also be linked with Walker’s finding among nurses in South Africa that used the term “terminating womanhood,” implying that women asking for an abortion were denying their role as reproductive agents [26]. With the increasing demands at both the individual and health system level for the spacing of pregnancies, combined with the still high unmet demands for contraception in the married population in Ethiopia [20], the limited acceptance for and access to safe abortion for married women emerges as ethically challenging. This is particularly so for poor, married women with very restricted control over their fertility.

### Religious and ethical justifications

In ethical dilemmas, ethical principles or values are at stake and will conflict with each other in a manner such that people may disagree on how to balance the principles and the final decision [27]. Often, ethical dilemmas arise when professional ethics or personal values require health-care workers to exercise liberal interpretations of the law [28]. Provision of abortion services seemed to activate core values and principles among study participants; removing or taking the life of a fetus was viewed by some people as doing harm, or maleficence, while others viewed assisting vulnerable women as doing good, or beneficence [27]. The latter principle was commonly given most weight, and most of the health-care workers interviewed would assist women to have an abortion even in cases where the woman was not legally eligible under the strictest interpretation of the law. These judgments emerge as manifestations of ‘wanting to do good’ scenarios. Trying to defend the ethical principles of ‘doing good’ and ‘avoiding harm’ nonetheless forced the health-care providers into moral grey zones, such that they accepted what they deemed to be lies and thus ‘stretched’ the law. The health workers’ responsibility to accept a woman’s word regardless of their own views of the situation imposed substantial burdens on them.

Rather than reflecting on abortion as an expression of women’s *autonomy* to decide over their bodies, abortion emerged in the health workers’ accounts as a *public health issue*, as a way of preventing the extreme health-related hazards of unsafe abortions, preventing young and poor unmarried women from giving birth, and allowing young women to complete their schooling. Other studies have shown how abortion service providers in African countries in a similar manner justify their provision of abortion services with the important public health impact of reducing the fatal consequences of unsafe abortion [9]. For example as Harries et al. found among nurses in South Africa who considered the potential socio-economic hardship a woman would face without an abortion as a sympathizing factor to justify abortions [29].

### Individual assessment and fairness concerns

The health-care workers in the study had a gatekeeping role regarding women’s actual access to safe abortion services and thus had substantial impact on who did and who did not get access to safe abortion. This situation led to some decisions being based more on the service provider’s personal values and notions of the legitimacy of women’s reasons for abortion thus creating discrimination and inequality in the access to safe abortion services*.* Studies indicate that when the distribution of scarce or ethically controversial treatment is left to clinicians without providing them with training, which includes reflection on values and controversies as well as careful and sufficient guidance, there may be unintended or unethical consequences [30]. However, providing health-care workers with the authority to apply the abortion law is not per definition ‘unfair’ if the law is legitimate and is properly understood and followed. As we have seen in the present study, this situation even provides health workers some room for individual assessment of the perceived need and the hazardous implications of refusing access.

### Moral distress

With the abortion law of 2005 and the guidelines of 2014, Ethiopian health-care workers were given explicit guidance for provision of safe abortion services. Several of the participants in our study stated that their role was experienced as a stressful burden which exhausted them. In line with descriptions of abortion service providers in South Africa, this stress was magnified by the stigmatization of their work and the feeling of being alone in their decision-making [29]. Similarly to the Yang et al. study about abortion service providers from Taiwan, our participants did not talk about their jobs with others, and they continuously argued with themselves about whether or not they acted against their religion [31]. Yang et al. describe how the “concealing of emotions toward abortion,” generated moral distress, described as doing something against one’s moral values [31]. This was similarly seen among Japanese midwives who expressed self-criticism and the hiding of emotions, which made it difficult for them to provide compassionate care during abortion services [32]. Moral distress can also occur when people act according to their morals but break the law in doing so. This has been described by Kälvemark et al. in their study of the ethical dilemmas in the Swedish healthcare system, where health workers gave medication for free to patients they knew would not be able to pay [33]. Similarly, in our study, many abortion service providers performed illegal abortions because they believed it was the best for the women, though they felt morally stressed about breaking the law. Other studies show how stress and negative attitudes among abortion service providers increase particularly if the abortion service providers feel uncomfortable with abortion and the law [8].

None of our study participants reported that they had regular ethical discussions at their institutions, and many carried their ethical struggles alone. The government in Ethiopia has recently launched a strategy to train a “Compassionate, Respectful and Caring Health Workforce,” [34]. The strategy is ambitious, aiming to reach and train all health workers in professionalism, ethics, communication, and legal regulations. This initiative is immensely valuable; however, far more initiatives are needed to ease the burden of the abortion service providers in a manner that simultaneously ensures that women wishing to obtain safe abortion are well and rightly cared for. Acknowledgement of the challenges the abortion service providers experience is vital. Support and mentoring should be continuously provided to the abortion service providers by their leaders and colleagues, and leaders should be made responsible for their crucial role in hindering bullying and distress among their employers.

### Strengths and limitations

A potential limitation to our study is that we only interviewed abortion service providers in the city of Addis Ababa. Moreover, assuming that ethics is colored by the context, experienced dilemmas and challenges are likely to vary, and hence one should be careful with generalizing our findings. Nonetheless, we believe that our study provides an important glimpse into the dilemmas that abortion service providers are likely to experience beyond Addis Ababa, as the law and the clinical guidelines regulating the field of abortion are the same throughout the country.

## Conclusion

In a country with a recently liberalized abortion law, the abortion service providers in Ethiopia seem to play a vital role in both enhancing women’s access to safe abortion and assessing if their reasons for abortion are acceptable or not. Various experienced ethical dilemmas, the moral distress of providing a service they perceive as lifesaving as well as sinful, and the stigma of being an abortion provider, leads to various personal dilemmas and coping mechanisms. To ensure both equal access and support for abortion-seeking women, and to equip the health workers with sufficient competence in making legitimate and ethically sound decisions, teaching and training programs in ethics, communication skills, and professionalism must be developed and implemented and support mechanisms and mentoring should be provided. Further research on abortion service providers roles and influence on abortion service provision should be conducted to inform policy aiming to reduce unsafe abortion and resultant deaths.

## Additional file

Additional file 1: Table of participants characteristics. The table shows in more detail the characteristics of the study participants giving information on gender, profession, years of working with abortion and religion. (DOCX 14 kb)

## Data Availability

The dataset generated and analysed during the current study are not publicly available due to confidentiality and protection of anonymity for the study participants.

## Abbreviations

FGD: Focus group discussion
ID: In-depth interview
MVA: Manual vacuum aspiration
NGO: Non-governmental organization
REK: Regional ethical committee
