# Supplementary material for: When the law makes doors slightly open: ethical dilemmas among abortion service providers in Addis Ababa, Ethiopia
Source: BMC Med Ethics. 2019 Sep 5;20:60. doi: 10.1186/s12910-019-0396-4 (PMC6727528; doi:10.1186/s12910-019-0396-4)
Supplement: Supplementary file 1 — Table of participants characteristics. The table shows in more detail the characteristics of the study participants giving information on gender, profession, years of working with abortion and religion. (DOCX 14 kb) [file 12910_2019_396_MOESM1_ESM.docx]

# **Additional file 1**

This section includes a table of information about the participants of our study. As one might notice not all the information is given for every participant, this is because abortion is a sensitive topic and it was not always appropriate to ask about personal information during the interviews. Also the age of the participants are not given in order to make sure that the study participants are properly anonymized.

**Table of participants characteristics:**

| **Study participants** | | | |
| --- | --- | --- | --- |
| **Sex** | **Profession** | **Years of working with abortion** | **Religion** |
| F | Nurse | 15 | Muslim |
| M | Nurse/Midwife | 2 | Orthodox |
| M | Nurse/Midwife | 2 1/2 | Christian |
| M | Nurse/Public health officer | 6 | Muslim |
| F | Doctor | 1 year and 1 month | Orthodox |
| F | Doctor | 6 months | Protestant |
| F | Doctor | More than 6 months | Orthodox |
| M | Nurse/Midwife | 4 | Protestant |
| M | Nurse | 1 | Orthodox |
| F | Nurse | 3 | Religious |
| M | Nurse/Midwife | 1 1/2 | Orthodox |
| MM | Medical students | Done abortions a few times | Orthodox |
| M | Medical students | Done abortions a few times | Religious |
| M | Doctor | 13 | Christian |
| M | Clinical officer/Doctor | 4 | Christian |
| F | Nurse | 2 |  |
| M | Nurse | 5 | Orthodox |
| M | Nurse | 8 months | Orthodox |
| F | Nurse | 3 years | Orthodox |
| F | Nurse/Midwife | 12 | Protestant |
| F | Nurse | 10 | Orthodox |
| F | Nurse | 2 | Orthodox |
| F | Nurse | 3 | Orthodox |
| M | Nurse/Midwife | 4 | Orthodox |
| F | Nurse and now medical student | Many years | Christian |
| M | Nurse/Midwife | 7 | Orthodox |
| M | Nurse | 2 | Orthodox |
| F | Nurse | 2 | Orthodox |
| F | Health officer | A bit over 2 | Religious |
| F | Working at abortion clinic, title unspecified | 5 | Religious |
| F | Working at abortion clinic, title unspecified | 6 | Religious |
| F | Working at abortion clinic, title unspecified | 7 |  |
| M | Working at abortion clinic, title unspecified |  | Religious |
| F | Nurse/midwife | 10 |  |
| F | Public health officer coordinating the clinic | 19 |  |
| F | Nurse/midwife | 15 |  |
| F | Nurse | 5 |  |
| M | Nurse | 12 |  |
| F | Nurse | 4 | Christian |
| F | Public health officer coordinating the clinic | 11 |  |
| M | Pharmacist | 2 |  |
